# A Novel Bat Algorithm Based on Differential Operator and Lévy Flights Trajectory

**DOI:** 10.1155/2013/453812

**Published:** 2013-03-17

**Authors:** Jian Xie, Yongquan Zhou, Huan Chen

**Affiliations:** ^1^College of Information Science and Engineering, Guangxi University for Nationalities, Nanning, Guangxi 530006, China; ^2^Guangxi Key Laboratory of Hybrid Computation and IC Design Analysis, Guangxi University for Nationalities, Nanning, Guangxi 530006, China

## Abstract

Aiming at the phenomenon of slow convergence rate and low accuracy of bat algorithm, a novel bat algorithm based on differential operator and Lévy flights trajectory is proposed. In this paper, a differential operator is introduced to accelerate the convergence speed of proposed algorithm, which is similar to mutation strategy “DE/best/2” in differential algorithm. Lévy flights trajectory can ensure the diversity of the population against premature convergence and make the algorithm effectively jump out of local minima. 14 typical benchmark functions and an instance of nonlinear equations are tested; the simulation results not only show that the proposed algorithm is feasible and effective, but also demonstrate that this proposed algorithm has superior approximation capabilities in high-dimensional space.

## 1. Introduction

Nowadays, since the evolutionary algorithm can solve some problem that the traditional optimization algorithm cannot do easy, the evolutionary algorithms are widely applied in different fields, such as the management science, engineering optimization, scientific computing. More and more modern metaheuristic algorithms inspired by nature or social phenomenon are emerging and they become increasingly popular, for example, particles swarms optimization (PSO) [1], firefly algorithm (FA) [2, 3], artificial chemical reaction optimization algorithm (ACROA) [4], glowworm swarms optimization (GSO) [5], invasive weed optimization (IWO) [6], differential evolution (DE) [7–9], bat algorithm (BA) [2, 10], and so on [11–15]. Some researchers have proposed their hybrid versions by combining two or more algorithms.

Bat Algorithm (BA) is a novel metaheuristic optimization algorithm based on the echolocation behaviour of microbats, which was proposed by Yang in 2010 [2, 10]. This algorithm gradually aroused people's close attention, and which is increasingly applied to different areas. Tsai et al. (2011) proposed an improved EBA to solve numerical optimization problems [16]. A multiobjective bat algorithm (MOBA) is proposed by Yang (2011) [17], which is first validated against a subset of test functions, and then applied to solve multiobjective design problems such as welded beam design. In 2012, Bora et al. applied BA to solve the Brushless DC Wheel Motor Problem [18]. Although the basic BA has remarkable property compared against several traditional optimization methods, the phenomenon of slow convergence rate and low accuracy still exists. Therefore, in this paper, we put forward an improved bat algorithm based on differential operator and Lévy flights trajectory (DLBA), the purpose is to improve the convergence rate and precision of bat algorithm. At the end of this paper, we tested 14 typical benchmark functions and applied them to solve nonlinear equations; the simulation results not only showed that the proposed algorithm is feasible and effective, which is more robust, but also demonstrated the superior approximation capabilities in high-dimensional space.

The rest of this study is organized as follows. In Section 2, the basic bat algorithm and Lévy flights were described. In Section 3, we gave the design framework of DLBA. The implementation and comparison of improved algorithm are presented in Section 4. Finally, we concluded this paper in Section 5.

## 2. Bat Algorithm, Lévy Flights, and Nonlinear Equations

### 2.1. Behaviour of Microbats

Most of microbats have advanced capability of echolocation. These bats can emit a very loud and short sound pulse; the echo that reflects back from the surrounding objects is received by their extraordinary big auricle. Then, this feedback information of echo is analyzed in their subtle brain. They not only can discriminate direction for their own flight pathway according to the echo, but also can distinguish different insects and obstacles to hunt prey and avoid a collision effectively in the day or night.

### 2.2. Bat Algorithm (see [10])

First of all, let us briefly review the basics of the BA for single-objective optimization. In the basic BA developed by Yang in 2010, in order to propose the bat algorithm inspired by the echolocation characteristics of microbats, the following approximate or idealised rules were used.

*IR1*. All bats use echolocation to sense distance, and they also “know” the difference between food/prey and background barriers in some magical way.

*IR2*. Bats fly randomly with velocity *v*_*i*_ at position *x*_*i*_ with a fixed frequency *f*_min⁡_, varying wavelength *λ*, and loudness *A*_0_ to search for prey. They can automatically adjust the wavelength (or frequency) of their emitted pulses and adjust the rate of pulse emission *r* ∈ [0,1], depending on the proximity of their target.

*IR3*. Although the loudness can vary in many ways, we assume that the loudness varies from a large (positive) *A*_0_ to a minimum constant value *A*_min⁡_.

In addition, for simplicity, they also use the following approximations: in general, the frequency *f* in a range [*f*_min⁡_, *f*_max⁡_] corresponds to a range of wavelengths [*λ*_min⁡_, *λ*_max⁡_]. In fact, they just vary in the frequency while fixed in the wavelength *λ* and assume *f* ∈ [0, *f*_max⁡_] in their implementation. This is because *λ* and *f* are related due to the fact that *λf* = *v* is constant. 

In simulations, they use virtual bats naturally to define the updated rules of their positions *x*_*i*_ and velocities *v*_*i*_ in a D-dimensional search space. The new solutions *x*_*i*_^*t*^ and velocities *v*_*i*_^*t*^ at time step *t* are given by

(1) $$\begin{array}{c} f_{i}=f_{\min}+\left(f_{\max}-f_{\min}\right)\beta ,\\ v_{i}^{t}=v_{i}^{t-1}+\left(x_{i}^{t}-x_{*}\right)f_{i},\\ x_{i}^{t}=x_{i}^{t-1}+v_{i}^{t}, \end{array}$$

where *β* ∈ [0,1] is a random vector drawn from a uniform distribution. Here, *x*_∗_ is the current global best location (solution) which is located after comparing all the solutions among all the *n* bats.

For the local search part, once a solution is selected among the current best solutions, a new solution for each bat is generated locally using random walk:

(2) $$\begin{array}{c} x_{\text{new}}=x_{\text{old}}+\varepsilon A_{t}, \end{array}$$

where *ε* ∈ [−1,1] is a random number, while *A*_*t*_ = 〈*A*_*i*_^*t*^〉 is the average loudness of all the bats at this time step. 

Furthermore, the loudness *A*_*i*_ and the rate *r*_*i*_ of pulse emission have to be updated accordingly as the iterations proceed. These formulas are

(3) $$\begin{array}{c} A_{i}^{t+1}=\alpha A_{i}^{t}, \end{array}$$

(4) $$\begin{array}{c} r_{i}^{t+1}=r_{i}^{0}\left[1-\exp\left(-\gamma t\right)\right], \end{array}$$

where *α* and *γ* are constants.

Based on these approximations and idealization, the basic steps of the bat algorithm can be summarized in the Pseudocode 1.

### 2.3. Lévy Flights

Lévy flights are Markov processes, which differ from regular Brownian motion, whose individual jumps have lengths that are distributed with the probability density function (PDF) *λ*(*x*) decaying at large *x* as *λ*(*x*) = |*x*|^−1−*α*^ with 0 < *α* < 2. Due to the divergence of their variance, *λ*(*x*)*≃x*|^−1−*α*^, extremely long jumps may occur, and typical trajectories are self-similar, on all scales showing clusters of shorter jumps interspersed by long excursions [19]. Lévy flight has the following properties [20]:

1. *Stability*: distribution of the sum of independent identically distributed stable random variables equal to distribution of each variable.
2. *Power law asymptotics* (“heavy tails”).
3. *Generalized Central Limit Theorem*: The central limit theorem states that the sum of a number of independent and identically distributed (i.i.d.) random variables with finite variances will tend to a normal distribution as the number of variables grows.
4. *Which has an infinite variance with an infinite mean value.*

Due to the these remarkable properties of stable distributions, it is now believed that the Lévy statistics provide a framework for the description of many natural phenomena in physical, chemical, biological, and economical systems from a general common point of view.

Furthermore, various studies have shown that the flight behaviour of many animals and insects has demonstrated the typical characteristics of Lévy lights. A recent study by Reynolds and Frye shows that fruit flies, or *Drosophila melanogaster*, explore their landscape using a series of straight flight paths punctuated by a sudden 90° turn, leading to a Lévy flight-style intermittent scale-free search pattern [21]. Studies on human behaviour such as the Ju/'hoansi hunter-gatherer foraging patterns also show the typical feature of Lévy flights [22]. The conclusion that light is related to Lévy flights is proposed by Barthelemy et al. (2008) [23]. The study by Mercadier et al. shows that the Lévy flights of photons in hot atomic vapours (2009) [24]. Subsequently, such behaviour has been applied to optimization and optimal search, and preliminary results show its promising capability.

### 2.4. Description of the Nonlinear Equations (see [25])

The general form of nonlinear equations with real variables is described as follows:

(5) $$\begin{array}{c} g_{i}\left(X\right)=0,\;i=1,2,\ldots ,n, \end{array}$$

where *X* = (*x*_1_, *x*_2_,…, *x*_*n*_) ∈ *D* ⊂ *R* ^*n*^, *D* = {(*x*_1_, *x*_2_,…, *x*_*n*_) | *a*_*i*_ ≤ *x*_*i*_ ≤ *b*_*i*_, *i* = 1,2,…, *n*}.

In solving nonlinear equations process for DLBA, the fitness function can be constructed by

(6) $$\begin{array}{c} G\left(X\right)=\sum_{i=1}^{n}\left|g_{i}\left(x\right)\right|. \end{array}$$

So, the solving of nonlinear equations can be translated into an optimization problem in domain *D*:

(7) min⁡⁡  G(X)s.t⁡  (x1,x2,…,xn)∈D.

Consequently, the optimal value of (7) is exactly the solution of (5).

## 3. DLBA

Inspired by Yang's method, we propose an improved bat algorithm based on differential operator and Lévy-flights trajectory (DLBA) based on the basic structure of BA and re-estimate the characters used in the original BA. In DLBA, not only the movement of the bat is quite different from the original BA, but also the local search process is different.

In DLBA, the frequency fluctuates up and down, which can change self-adaptively, and the differential operator is introduced, which is similar to the mutation operation of DE, the frequency *f* is similar to the scale factor *F* of DE/best/2. So, the frequency updated formulae of a bat are defined as follows:

(8) $$\begin{array}{c} f_{1i}^{t}=\left(\left(f_{1,\min}-f_{1,\max}\right)\frac{t}{n_{t}}+f_{1,\max}\right)\beta_{1}, \end{array}$$

(9) $$\begin{array}{c} f_{2i}^{t}=\left(\left(f_{2,\max}-f_{2,\min}\right)\frac{t}{n_{t}}+f_{2,\min}\right)\beta_{2}, \end{array}$$

where *β*_1_, *β*_2_ ∈ [0,1] is a random vector drawn from a uniform distribution, *f*_1,max⁡_ = *f*_2,max⁡_, *f*_1,min⁡_ = *f*_2,min⁡_, *n*_*t*_ is a fixed parameter. In DLBA, the position*x*_*i*_^*t*^of each bat individual are updated with (10), which is different from original BA. This can preferably incorporate the echolocation characteristics of microbats:

(10) $$\begin{array}{c} x_{i}^{t+1}=x_{\text{best}}^{t}+f_{1i}^{t}\left(x_{r1}^{t}-x_{r2}^{t}\right)+f_{2i}^{t}\left(x_{r3}^{t}-x_{r4}^{t}\right), \end{array}$$

where *x*_best_^*t*^ is the current global best location (solution) which is located after comparing all the solutions among all the *n* bats in *t* generation, *x*_*ri*_^*t*^ is the bat individual in the bat swarm, and this can be achieved by randomization.

In addition, Lévy flight haves the prominent properties increase the diversity of population, sequentially, which can make the algorithm effectively jump out of the local optimum. So, we let these bats perform the Lévy flights with (11) before the position updating:

(11) $$\begin{array}{c} x_{i}^{t}={\hat{x}}_{i}^{t-1}+\mu \mathrm{sign}\left[\text{rand}-0.5\right]⊕\text{Levy}, \end{array}$$

where *μ* is a random parameter drawn from a uniform distribution, sign ⊕ means entrywise multiplications, rand ∈ [0,1], and random step length Levy obeys Lévy distribution:

(12) $$\begin{array}{c} \text{Levy}\sim u=t^{-\lambda },\;\left(1<\lambda \leq 3\right). \end{array}$$

On the other hand, each bat should have different values of loudness and pulse emission rate, while the rate of pulse emission is relatively low and the loudness is relatively high. During the search process, the loudness usually decreases, while the rate of pulse emission increases. Bats' position variation is influenced by the pulse emission rate and loudness as well. Firstly, pulse emission rate *r*_*i*_ causes fluctuation of position using (2); sequentially, more and more new position can be explored. Secondly, loudness *A*_*i*_ is designed to strengthen local search and to guide bats find better solutions:

(13) $$\begin{array}{c} x_{\text{new}}=x_{\text{best}}+\eta r_{t}, \end{array}$$

where *η* ∈ [−1,1] is a random parameter, and *x*_best_ is the current global best location (solution) in whole bats swarm. While *r*_*t*_ = 〈*r*_*i*_^*t*^〉 is the average pulse emission rate of all the bats at this generation. 

The new rate of pulse emission *r*_*i*_^*t*^ and loudness *A*_*i*_^*t*^ at time step *t* are given by

(14) $$\begin{array}{c} r_{i}^{t+1}=r_{i}^{t}{\left(\frac{t}{n_{t}}\right)}^{3}, \end{array}$$

where *r*_*i*_^*t*^ is time varying; their loudness and emission rates will be updated only if the best solution of the current generation is better than the best solution of last generation, which means that these bats are moving towards the optimal solution. The pseudocode of DLBA can be depicted as in Pseudocode 2.

## 4. The Simulation and Analysis

### 4.1. Parametric Studies

The proposed DLBA is implemented in MATLAB, simulation platform: CPU Intel Xeon E5405@2.00 GHz; OS: Microsoft Windows Server 2003 Enterprise Edition SP2; RAM: 1 GHz; Matlab Version: R2009a. The parameter setting for BA and DLBA are listed in Table 1, the parameters of BA are recommended in the original article.

The stopping criterion can be defined in many ways. We adopt two terminated criteria: we can use a given tolerance (Tol = 1.0*e* − 5) for a test function that have certain minimum value in theory; on the contrary, each simulation run terminates when a certain number of function evaluations (FEs) have been reached. In this paper, FEs could be obtained by population size multiplied by the number of iteration. In our experiment, FEs = 24000, BA (= 300∗40∗2); DLBA (= 200∗40∗3).

### 4.2. Benchmark Test Function

In order to validate the validity of DLBA, we selected the 14 benchmark functions to experimentize. The benchmark set include unimodal, multimodal, high-dimensional, and low-dimensional unconstrained optimization benchmark functions, where *f*_1_–*f*_3_ are unimodal functions, *f*_4_–*f*_14_ are multimodal functions; *f*_1_–*f*_9_ have certain theoretical minimum and *f*_10_–*f*_14_ have uncertain theoretical minimum.

(1)  *f*_1_: sphere function (the first function of De Jong's test set),

(15) $$\begin{array}{c} f\left(x\right)=\sum_{i=1}^{n}x_{i}^{2},\;-10\leq x_{i}\leq 10. \end{array}$$

Here, *n* is dimensionality; this function has a global minimum *f*_min⁡_ = 0 at *x*_∗_ = (0,0,…, 0).

(2)  *f*_2_: Schwegel's problem 2.22,

(16) $$\begin{array}{c} f\left(x\right)=\sum_{i=1}^{n}\left|x_{i}\right|+\prod_{i=1}^{n}\left|x_{i}\right|,\;-10\leq x_{i}\leq 10, \end{array}$$

whose global minimum is obviously *f*_min⁡_ = 0 at *x*_∗_ = (0,0,…, 0).

(3)  *f*_3_: Rosenbrock function,

(17) f(x)=∑i=1n−1[(xi−1)2+100(xi+1−xi2)2],           −2.408≤xi≤2.408,

which has a global minimum *f*_min⁡_ = 0 at *x*_∗_ = (1,1,…, 1).

(4)  *f*_4_: Eggcrate function,

(18) f(x,y)=x2+y2+25(sin2x+sin2y),          (x,y)∈[−2π,2π].

This 2-dimensional test function obviously gets the global minimum *f*_min⁡_ = 0 at (0, 0).

(5)  *f*_5_: Ackley's function,

(19) f(x)=−20exp⁡[−151n∑i=1nxi2]    −exp⁡[1n∑i=1ncos⁡⁡(2πxi)]+20+e,               −30≤x≤30.

This function has a global minimum *f*_min⁡_ = 0 at *x*_∗_ = (0,0,…, 0), which is a multimodal function.

(6)  *f*_6_: Griewangk's function,

(20) $$\begin{array}{c} f\left(x\right)=\frac{1}{4000}\sum_{i=1}^{n}x_{i}^{2}-\prod_{i=1}^{n}\cos\left(\frac{x_{i}}{\sqrt{i}}\right)+1,\;-600\leq x_{i}\leq 600. \end{array}$$

Its global minimum equal *f*_min⁡_ = 0 is obtainable for *x*_∗_ = (0,0,…, 0), the number of local minima for arbitrary *n* is unknown, but in the two-dimensional case there are some 500 local minima.

(7)  *f*_7_: Salomon's function,

(21) $$\begin{array}{c} f\left(x\right)=-\cos\left(2\pi \sqrt{\sum_{i=1}^{n}x_{i}^{2}}\right)+0.1\sqrt{\sum_{i=1}^{n}x_{i}^{2}}+1,\;-5\leq x_{i}\leq 5, \end{array}$$

which has a global minimum *f*_min⁡_ = 0 at *x*_∗_ = (0,0,…, 0), and is a multimodal function.

(8)  *f*_8_: Rastrigin's function.

(22) $$\begin{array}{c} f\left(x\right)=10n+\sum_{i=1}^{n}\left[x_{i}^{2}-10\cos\left(2\pi x_{i}\right)\right],\;-5.12\leq x_{i}\leq 5.12, \end{array}$$

whose global minimum is *f*_min⁡_ = 0 at *x*_∗_ = (0,0,…, 0); for *n* = 2, there are about 50 local minimizers arranged in a lattice-like configuration.

(9)  *f*_9_: Zakharov's function,

(23) $$\begin{array}{c} f\left(x\right)=\sum_{i=1}^{n}x_{i}^{2}+{\left(\frac{1}{2}\sum_{i=1}^{n}ix_{i}\right)}^{2}+{\left(\frac{1}{2}\sum_{i=1}^{n}ix_{i}\right)}^{4},\;-10\leq x_{i}\leq 10, \end{array}$$

whose global minimum is *f*_min⁡_ = 0 at *x*_∗_ = (0,0,…, 0); it is a multimodal function as well.

(10)  *f*_10_: Easom's function,

(24) f(x,y)=−cos⁡(x)cos⁡(y)exp⁡[−(x−π)2+(y−π)2],                     −10≤x,  y≤10,

whose global minimum is *f*_min⁡_ = −1 at (*π*, *π*); it has many local minima. 

(11)  *f*_11_: Schwegel's function,

(25) $$\begin{array}{c} f\left(x\right)=-\sum_{i=1}^{n}x_{i}\sin\left(\sqrt{\left|x_{i}\right|}\right),\;-500\leq x_{i}\leq 500, \end{array}$$

whose global minimum is *f*_min⁡_ ≈ −418.9829*n* occuring at *x*_∗_ ≈ (420.9687,…, 420.9687).

(12)  *f*_12_: Shubert's function,

(26) f(x,y)=[∑i=15icos⁡(i+(i+1)x)]·[∑i=15icos⁡(i+(i+1)y)],                     −10≤x,  y≤10.

The number of local minima for this problem is not known but for *n* = 2, the function has 760 local minima, 18 of which are global with *f*_min⁡_ ≈ −186.7309.

(13)  *f*_13_: Xin-She Yang's function,

(27) $$\begin{array}{c} f\left(x\right)=-\left(\sum_{i=1}^{n}\left|x_{i}\right|\right)\exp\left(-\sum_{i=1}^{n}x_{i}^{2}\right),\;-10\leq x_{i}\leq 10, \end{array}$$

which has multiple global minima, for example, for *n* = 2, it has 4 equal minima $f_{\min}=-1/\sqrt{e}\approx -0.6065$ at (0.5, 0.5), (0.5-0.5), (−0.5, 0.5), and (−0.5, −0.5).

(14)  *f*_14_: “Drop Wave” function,

(28) $$\begin{array}{c} f\left(x\right)=-\frac{1+\cos\left(12\sqrt{\sum_{i=1}^{n}x_{i}^{2}}\right)}{\left(1/2\right)\left(\sum_{i=1}^{n}x_{i}^{2}\right)+2},\;-5.12\leq x_{i}\leq 5.12. \end{array}$$

This two-variable function is a multimodal test function, whose global minimum is *f*_min⁡_ = −1 occurs at *x*_∗_ = (0,0,…, 0).

### 4.3. Comparison of Experimental Results

**Figure 1:**
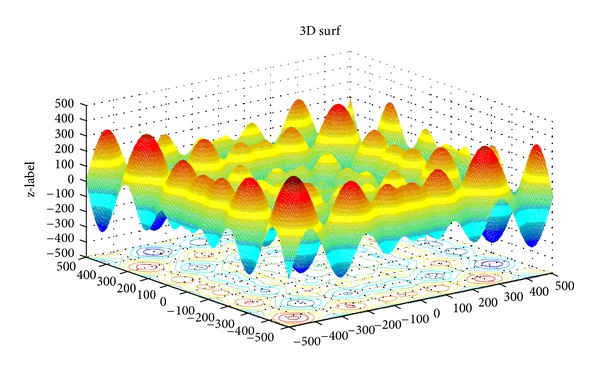
The landscape of Schwegel's function.

**Figure 2:**
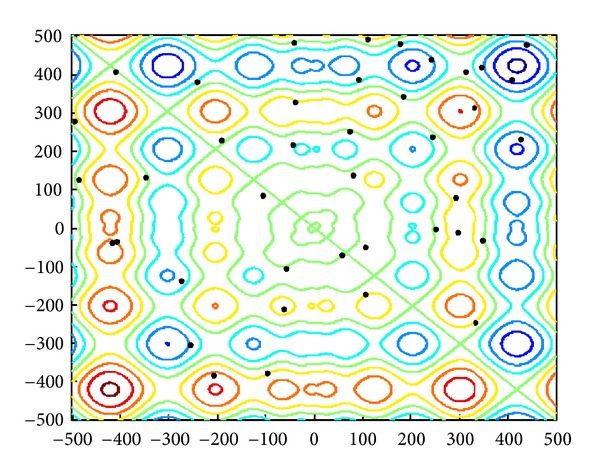
The locations of 40 bats in the initial phase.

**Figure 3:**
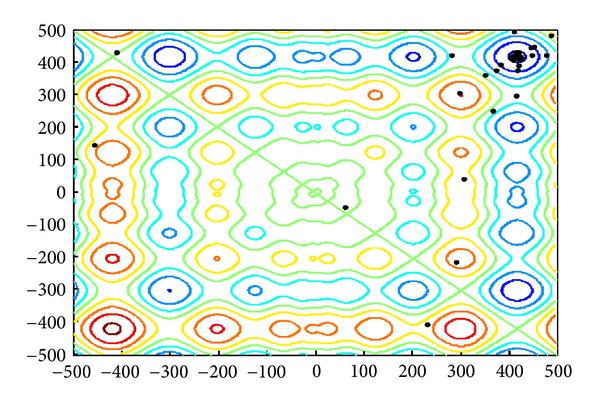
The locations of 40 bats after six iterations.

**Figure 4:**
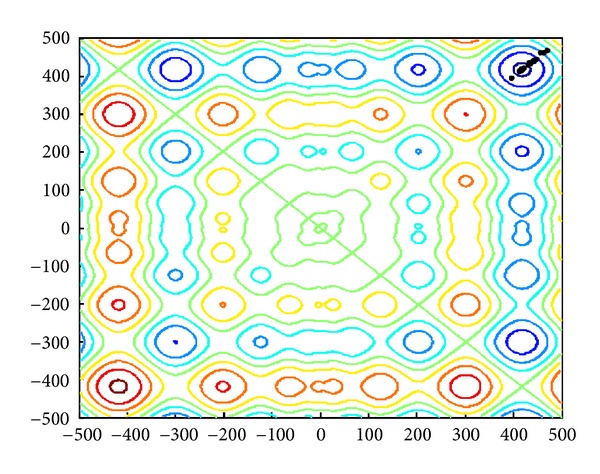
The locations of 40 bats after fifteen iterations.

The 2D landscape of Schwegel's function is shown in Figure 1, and this global minimum can be found after about 720 FEs for 40 bats after 6 iterations as shown in Figures 2, 3, and 4.

We adopt different terminated criteria aiming at different benchmark function, we perform 100 times independently for each test function, and the record is given in Tables 2 and 3. 

From Table 2, we can see that the DLBA performs much better than the basic bat algorithm, which converges much faster than BA under the fixed accuracy Tol = 1.0*e* − 5. In our experiment, for the function *f*_4_, we proposed that an algorithm only costs 6 generations under the best situation, and the average generations is 10. Furthermore, for the first group of test functions *f*_1_–*f*_9_, the DLBA averagely expend 43.9 generations when each function attains its terminated criteria; however, the BA needs 200 generations invariably, and the convergence speed of DLBA advances 298.57% times. On other hand, the DLBA which obtained the accuracy of the solution is much superior to BA, which is more approximate to the theoretical value. In a word, it demonstrated that DLBA has fast convergence rate and high precision of the solutions.

In Table 3, the five functions (*f*_10_–*f*_14_) independently runs 100 times under the 24000 Fes; we can clearly see the precision of DLBA is obviously superior to the bat algorithm. Some benchmark functions can easily attain the theoretical optimal value. In addition, the standard deviation of DLBA is relatively low. It shows that DLBA has superior approximation ability.

**Figure 5:**
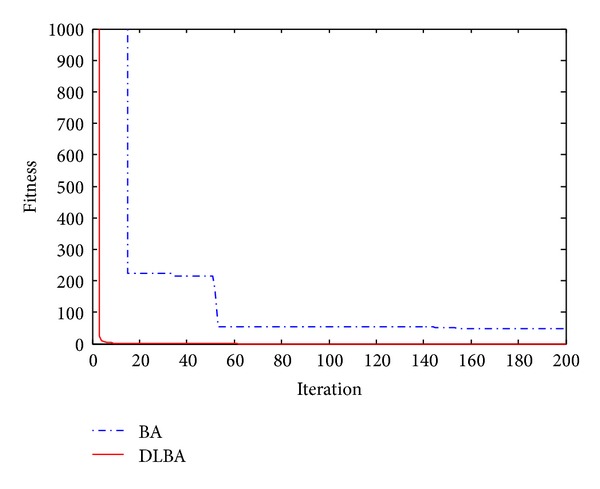
Convergence curves for the function *f*_2_.

**Figure 6:**
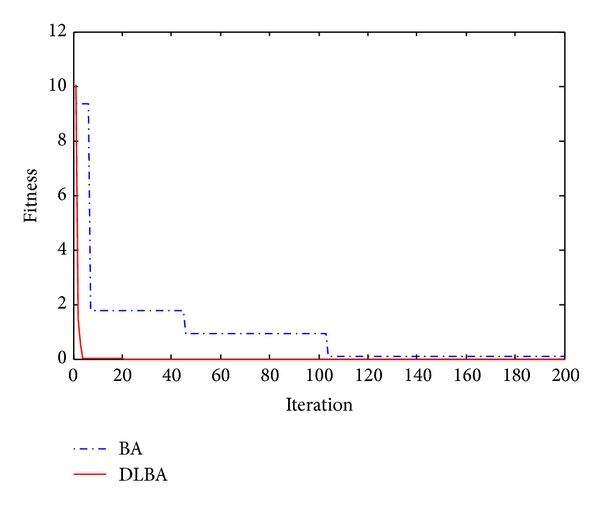
Convergence curves for the function *f*_4_.

**Figure 7:**
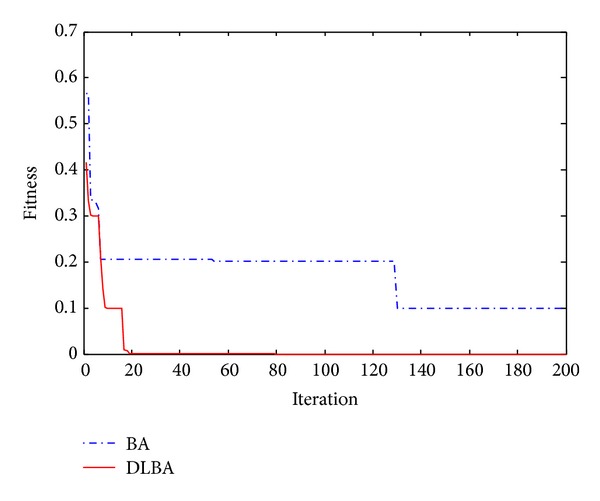
Convergence curves for the function *f*_7_.

**Figure 8:**
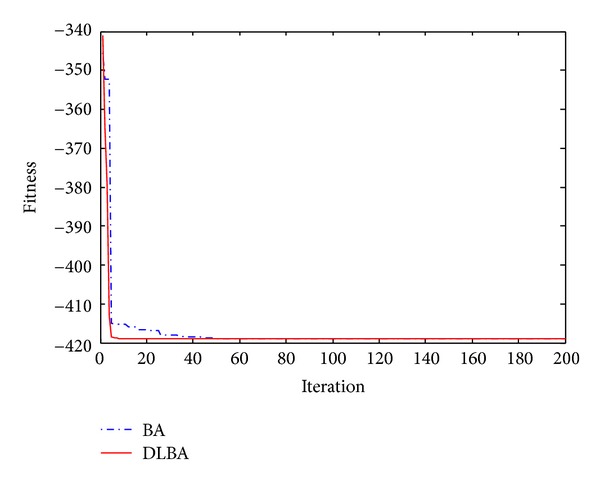
Convergence curves for the function *f*_11_.

**Figure 9:**
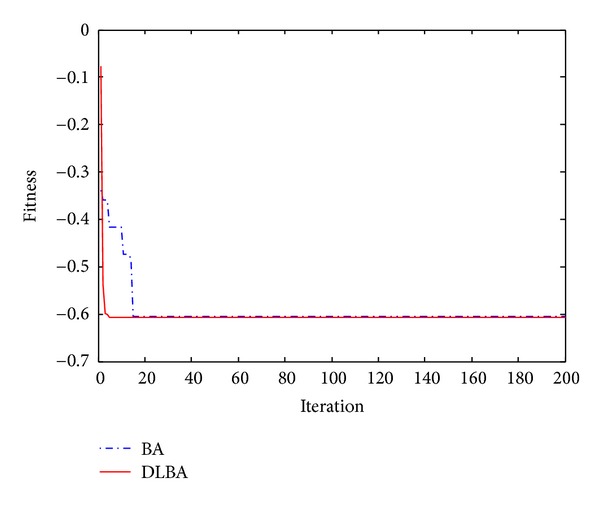
Convergence curves for the function *f*_13_.

Observe Tables 2 and 3, no matter high-dimensional or low-dimensional, we can find that DLBA can quickly converge to the global minima. Furthermore, Figures 5, 6, 7, 8, and 9 show the convergence curves for some of the functions from a particular run of DLBA and BA, which end at 200 generations. The adaptive scheme generally converges faster than the basic scheme. Here, we select *f*_2_ (*D* = 20), *f*_4_ (*D* = 2), *f*_7_ (*D* = 5), *f*_11_ (*D* = 2), and *f*_13_ (*D* = 2).

DLBA not only has superior approximation ability in low-dimensional space, but also has excellent global search ability in high-dimensional situation.  Table 4 is the experimental result that DLBA performs 50 times independently under the high-dimensional situation. As shown in Table 4, we can be conscious that the DLBA is effective under the multidimensional condition, and acquired solution has higher accuracy, even approximate the theoretical value.

**Figure 10:**
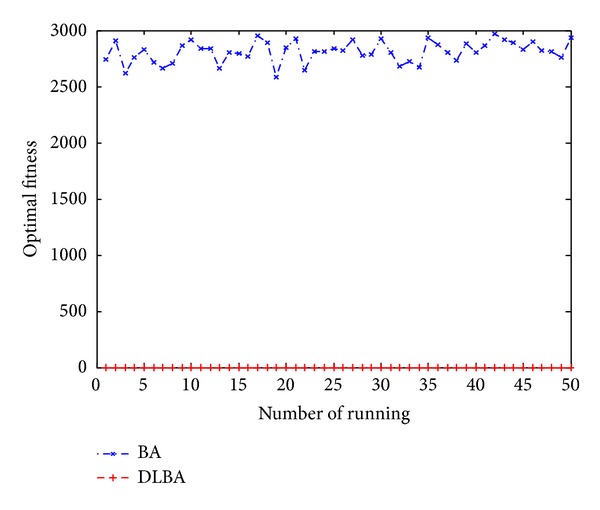
Distribution of optimal fitness for *f*_6_ (*D* = 128).

**Figure 11:**
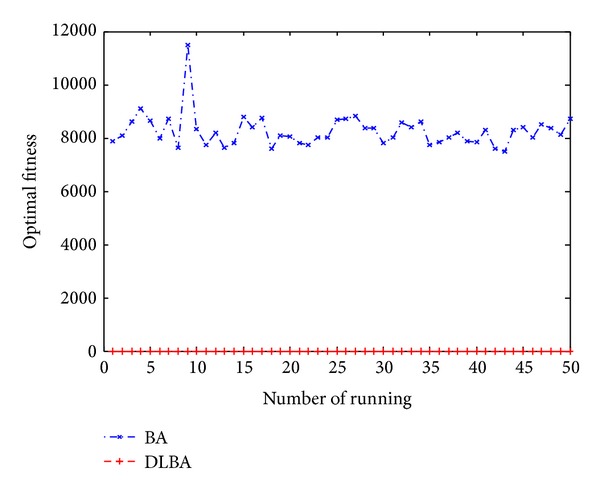
Distribution of optimal fitness for *f*_9_ (*D* = 256).

**Figure 12:**
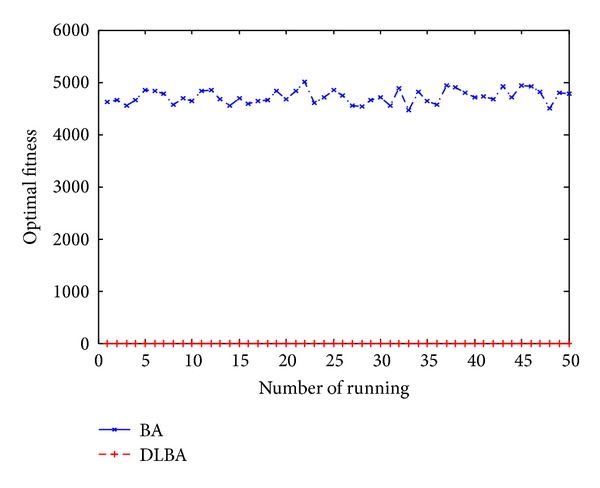
Distribution of optimal fitness for *f*_8_ (*D* = 320).

**Figure 13:**
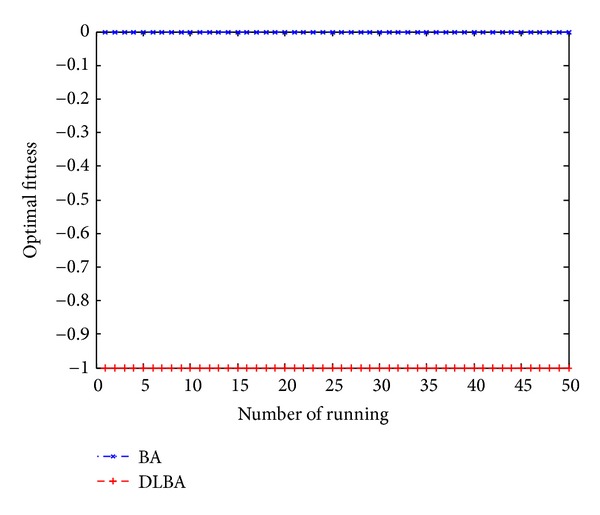
Distribution of optimal fitness for *f*_14_ (*D* = 512).

**Figure 14:**
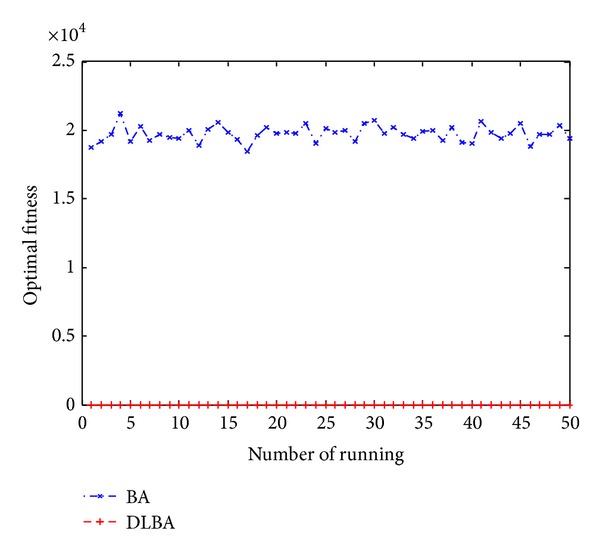
Distribution of optimal fitness for *f*_1_ (*D* = 1024).

**Figure 15:**
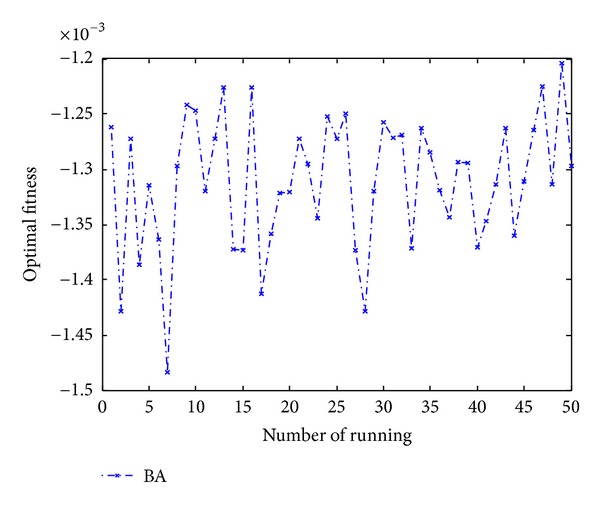
Details of BA line in Figure 12.

**Figure 16:**
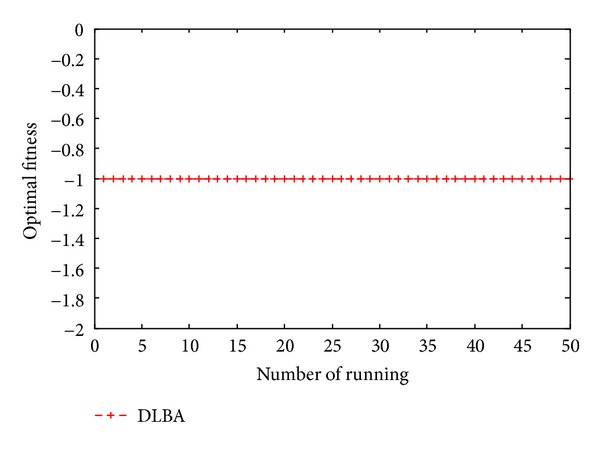
Details of DLBA line in Figure 12.

Figures 10, 11, 12, 13, and 14 are the distribution map of optimal fitness; that is the selected functions independently perform 50 times under the multidimensional situation.  Figure 13 show that two “straight line”, we can see in the figure that DLBA reaches the global optimum(−1), however, BA fluctuates around 0. In order to display the fact of fluctuation, we magnify the two “straight line”, and the amplifying effect are depicted in Figures 15 and 16. According to the experimental results which are obtained from selected test functions, DLBA presents higher precision than the original BA on minimizing the outcome as the optimization goal. 

### 4.4. An Application for Solving Nonlinear Equations

*Interval Arithmetic Benchmark (IAB)*. We consider one benchmark problem proposed from interval arithmetic, the benchmark consists of the following system of [25]:

(29) $$\begin{array}{c} 0=x_{1}-0.25428722-0.18324757x_{4}x_{3}x_{9},\\ 0=x_{2}-0.37842197-0.16275449x_{1}x_{10}x_{6},\\ 0=x_{3}-0.27162577-0.16955071x_{1}x_{2}x_{10},\\ 0=x_{4}-0.19807914-0.15585316x_{7}x_{1}x_{6},\\ 0=x_{5}-0.44166728-0.19950920x_{7}x_{6}x_{3},\\ 0=x_{6}-0.14654113-0.18922793x_{8}x_{5}x_{10},\\ 0=x_{7}-0.42937161-0.21180486x_{2}x_{5}x_{8},\\ 0=x_{8}-0.07056438-0.17081208x_{1}x_{7}x_{6},\\ 0=x_{9}-0.34504906-0.19612740x_{10}x_{6}x_{8},\\ 0=x_{10}-0.42651102-0.21466544x_{4}x_{8}x_{1}. \end{array}$$

**Figure 17:**
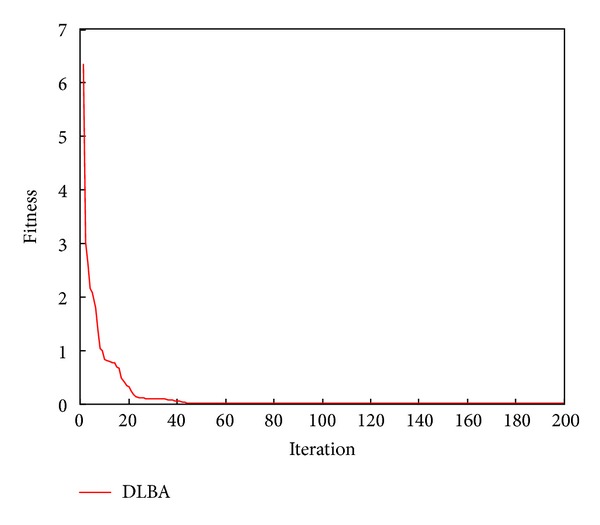
Convergence curves for DLAB.

**Figure 18:**
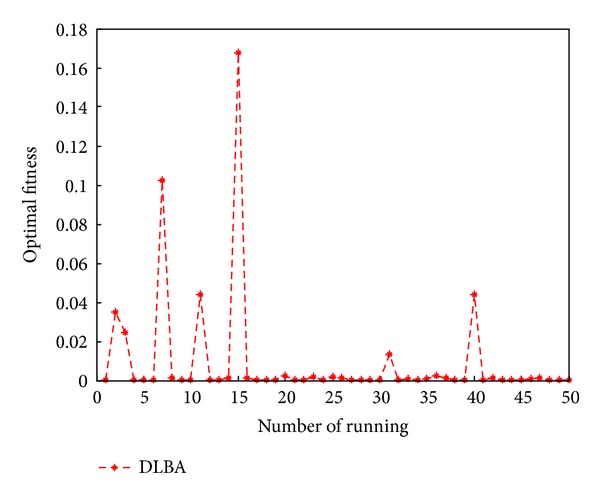
Distribution of optimal fitness for DLAB.

**Figure 19:**
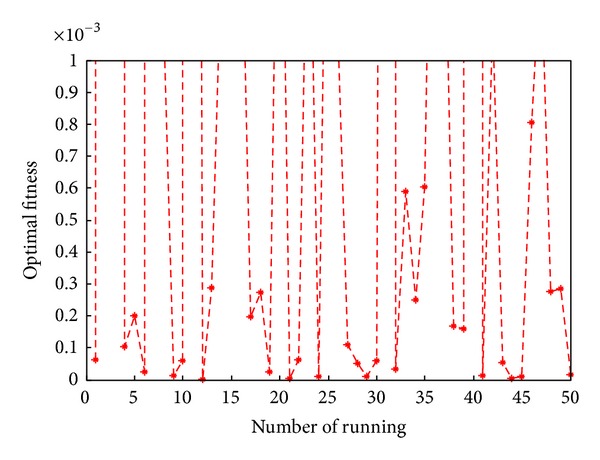
Distribution of optimal fitness less than 0.001 in Figure 18.

Some of the solutions obtained as well as the function values (which represent the values of the system's equations obtained by replacing the variable values) are presented in Table 5. From Table 5, we can obviously observe that the function value of each equation solved by DLBA is superior to which solved by EA [25], depending on the statistics of the 40 function values, the precision of function values is enhanced 5.199820*E* + 06 times by DLBA. The convergence curve of DLBA is depicted in Figure 17. Figure 17 show that DLBA has fast convergence rate for solving nonlinear equations. The achieved optimal fitness (the sum of function value with absolute value) in 50 times independent run is depicted in Figure 18. In order to reflect the precision of fitness, we magnify Figure 18, these optimal fitness that less than or equal to 0.001 are plotted in Figure 19. We can found that there are 32 times optimal fitness is less than 0.001.

## 5. Conclusions

Aiming at the phenomenon of slow convergence rate and low accuracy of bat algorithm, we put forward animproved bat algorithm with differential operator and Lévy flights trajectory (DLBA) based on the basic framework of bat algorithm (BA), the purpose is to improve the convergence rate and precision of bat algorithm. In this paper, we define the frequency fluctuations up and down when the bat tracking prey, which influence the bats' location problem; it is more graphic to simulate the bat's behavior. Moreover, it can make the algorithm effectively jump out of the local optimum to add Lévy flights and differential operator, the main reason is because Lévy flight has prominent properties in the previously mentioned and the differential operator can guide bats find better solutions, sequentially, increase the convergent speed.

In addition, bats' position variation is influenced by the pulse emission rate and loudness as well. Firstly, pulse emission rate causes update of position, and more and more new position can be explored, consequently, increasing the diversity of population. Secondly, loudness is designed to strengthen local search and to guide bats find better solutions.

In this paper, we tested 14 typical benchmark functions and applied them to solve nonlinear equations, the simulation results not only showed that the proposed algorithm is feasible and effective, which is more robust, but also demonstrated the superior approximation capabilities in high-dimensional space. This is not surprising as the aim of developing the new algorithm was to try to use the advantages of existing algorithms and other interesting feature inspired by the fantastic behavior of echolocation of microbats. Numerically speaking, these can be translated into two crucial characteristics of the modern metaheuristics: intensification and diversification. Intensification intends to search around the current best solutions and select the best candidates or solutions, while diversification makes sure that the algorithm can explore the search space efficiently. 

This potentially powerful optimization strategy can easily be extended to study multiobjective optimization applications with various constraints, even to NP-hard problems. Further studies can focus on the sensitivity and parameter studies and their possible relationships with the convergence rate of the algorithm.

## Figures and Tables

**Pseudocode 1:**
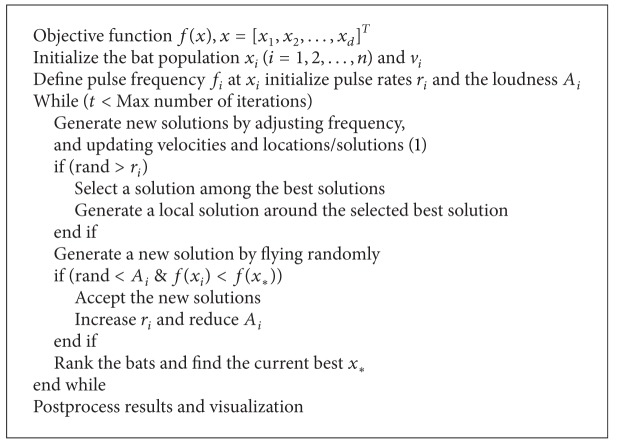


**Pseudocode 2:**
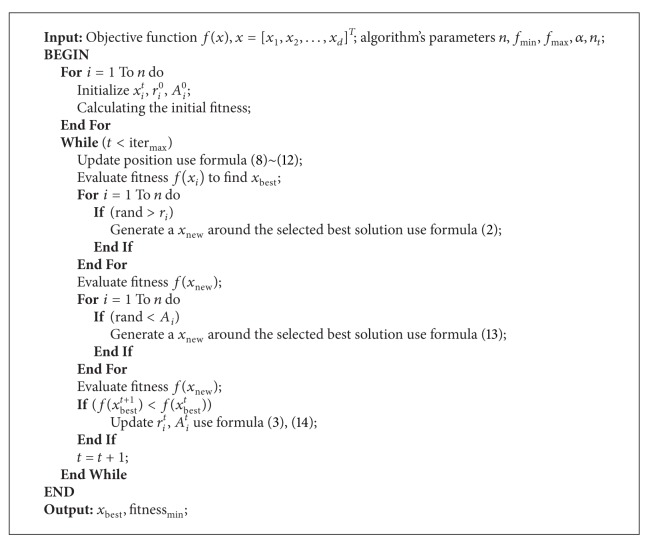


**Table 1:** The parameter set of BA and DLBA.

Algorithms	*n*	*f*_min⁡_	*f*_max⁡_	*A*_*i*_^0^	*r*_*i*_^0^	*α*	*γ*	*n*_*t*_
BA	40	0	100	(1,2)	(0,0.1)	0.9	0.9	—
DLBA	40	0	1	(1,2)	(0,0.1)	0.9	—	5000

**Table 2:** Comparison of BA and DLBA for several test functions under the first terminated criteria.

TC	BF	Method	Fitness				Iteration		
			Min	Mean	Max	Std	Min	Mean	Max
Tol = 1.0*e* − 5, iter_max_ = 200	*f*_1_ (*D* = 30)	DLBA	4.30568008*E* − 08	4.80356915*E* − 06	9.96163402*E* − 06	3.02060716*E* − 06	10	17.6	26
		BA	56.81732462	130.61959271	223.82694528	37.22960351	200	200	200
	*f*_2_ (*D* = 20)	DLBA	5.66761254*E* − 07	6.33091639*E* − 06	9.88929757*E* − 06	2.48867687*E* − 06	21	29.4	37
		BA	22.77136624	93.35062167	2733.46090499	269.41410301	200	200	200
	*f*_3_ (*D* = 10)	DLBA	5.66716240	7.39029403	8.28857171	0.37001430	200	200	200
		BA	82.24908050	294.95948479	640.76052284	109.67161818	200	200	200
	*f*_4_ (*D* = 2)	DLBA	6.23475433*E* − 09	3.93352701*E* − 06	9.97022879*E* − 06	2.77208752*E* − 06	6	10	20
		BA	1.05265280*E* − 04	0.20705750	1.00771980	0.23116047	200	200	200
	*f*_5_ (*D* = 5)	DLBA	4.71737180*E* − 07	6.40969812*E* − 06	9.96088247*E* − 06	2.71187009*E* − 06	16	25.6	39
		BA	1.15883977	3.02092450	5.86431360	0.69052427	200	200	200
	*f*_6_ (*D* = 5)	DLBA	1.38176777*E* − 07	5.01027883*E* − 06	9.92998256*E* − 06	2.77338474*E* − 06	11	18.4	56
		BA	0.06234957	8.42851961	29.90707979	6.12812390	200	200	200
	*f*_7_ (*D* = 5)	DLBA	7.78490674*E* − 07	6.57288799*E* − 06	9.97486019*E* − 06	2.36637154*E* − 06	18	46	114
		BA	0.09987344	0.15318115	0.30215065	0.04923496	200	200	200
	*f*_8_ (*D* = 5)	DLBA	5.99983281*E* − 08	4.47052788*E* − 06	9.85695441*E* − 06	2.81442645*E* − 06	15	33	87
		BA	7.24547772	17.11077150	25.90638177	4.37190073	200	200	200
	*f*_9_ (*D* = 5)	DLBA	6.65070016*E* − 09	3.88692603*E* − 06	9.69841027*E* − 06	2.75444981*E* − 06	10	15.1	23
		BA	0.11106843	1.75623424	8.48211975	1.41343722	200	200	200

**Table 3:** Comparison of BA and DLBA for several test functions under the second terminated condition.

TC	BF	Method	Fitness			
			Min	Mean	Max	Std
FEs	*f*_10_ (*D* = 2)	DLBA	−1	−1	−1	9.06493304*E* − 17
		BA	−0.99964845	−0.98245546	−0.90221232	0.01759602
	*f*_11_ (*D* = 2)	DLBA	−418.98288727	−415.83523725	−300.54455266	15.53948263
		BA	−418.98287874	−406.98044144	−328.10751671	22.54615132
	*f*_12_ (*D* = 5)	DLBA	−186.73090883	−186.73090883	−186.73090883	4.31613544*E* − 13
		BA	−186.67541533	−184.01677767	−174.95599301	2.26333511
	*f*_13_ (*D* = 2)	DLBA	−0.60653066	−0.60653066	−0.60653066	8.63947249*E* − 16
		BA	−0.60652631	−0.60411559	−0.59656608	2.18888608*E* − 03
	*f*_14_ (*D* = 5)	DLBA	−1	−1	−1	0
		BA	−0.93624514	−0.80454167	−0.56977584	0.10270830

**Table 4:** Comparison of DLBA and BA under the high-dimensional situation.

BF	Method	Fitness			
		Min	Mean	Max	Std
*f*_6_ (*D* = 128)	DLBA	0	0	0	0
	BA	2587.38759264	2811.40649000	2966.56723398	93.96861133
*f*_9_ (*D* = 256)	DLBA	2.66885939*E* − 94	5.12093542*E* − 69	2.56023720*E* − 67	3.62071553*E* − 68
	BA	7503.39306546	8256.61302714	11506.79192998	613.37556609
*f*_8_ (*D* = 320)	DLBA	0	0	0	0
	BA	4468.59039467	4723.44624200	5008.57109760	131.18818777
*f*_14_ (*D* = 512)	DLBA	−1	−1	−1	0
	BA	−1.48358700*E* − 03	−1.31084173*E* − 03	−1.20435493*E* − 03	5.99288716*E* − 05
*f*_1_ (*D* = 1024)	DLBA	2.63790469*E* − 109	4.73742970*E* − 87	2.03861481*E* − 85	2.89031313*E* − 86
	BA	18463.48950157	19752.26686813	21239.23111285	567.27878361

**Table 5:** Outcome of DLBA for Interval Arithmetic Benchmark And in Comparison with EA.

Method	EA		DLBA	
Solutions	Variables values	Functions values	Variables values	Functions values
Sol. 1	0.0464905115	0.2077959240	0.2578335208	7.22997083*E* − 08
	0.1013568357	0.2769798846	0.3810968901	−2.84879905*E* − 07
	0.0840577820	0.1876863212	0.2787447331	−2.63974619*E* − 07
	−0.1388460309	0.3367887114	0.2006720536	3.06212480*E* − 06
	0.4943905739	0.0530391321	0.4452522319	7.74692656*E* − 07
	−0.0760685163	0.2223730535	0.1491853777	1.33597657*E* − 06
	0.2475819110	0.1816084752	0.4320098178	−7.87244534*E* − 09
	−0.0170748156	0.0878963860	0.0734062202	3.41255029*E* − 06
	0.0003667535	0.3447200366	0.3459672005	3.24064555*E* − 07
	0.1481119311	0.2784227489	0.4273251429	−1.18429644*E* − 06

Sol. 2	0.1224819761	0.1318552790	0.2578332642	−1.45675448*E* − 07
	0.1826200685	0.1964428361	0.3810968252	−3.41069930*E* − 07
	0.2356779803	0.0364987069	0.2787462357	1.19723259*E* − 06
	−0.0371150470	0.2354890155	0.2006687659	−1.76074071*E* − 08
	0.3748181856	0.0675753064	0.4452514819	2.89773434*E* − 07
	0.2213311341	0.0739986588	0.1491839999	9.16571896*E* − 08
	0.0697813043	0.3607038292	0.4319795315	−3.01421570*E* − 05
	0.0768058043	0.0059182979	0.0734021258	−4.53845535*E* − 07
	−0.0312153867	0.3767487763	0.3459672388	4.15572858*E* − 07
	0.1452667120	0.2811693568	0.4273281275	1.85998012*E* − 06

Sol. 3	0.0633944399	0.1908436653	0.2578328072	−5.95667763*E* − 07
	0.1017426933	0.2767897367	0.3810969084	−2.39480772*E* − 07
	−0.1051842285	0.3769063436	0.2787447816	−2.04170456*E* − 07
	−0.0477059943	0.2460900702	0.2006696507	6.71241513*E* − 07
	0.4149858326	0.0260337751	0.4452514467	−4.38810984*E* − 09
	0.1215195321	0.0256054760	0.1491841089	1.89336147*E* − 07
	0.2539777159	0.1761486401	0.4320126793	2.97845790*E* − 06
	0.0843972823	0.1349869851	0.0734028724	7.79163472*E* − 08
	−0.0534132992	0.3986395691	0.3459668311	3.30962522*E* − 09
	0.0880998746	0.3383563536	0.4273256312	−6.46813249*E* − 07

Sol. 4	0.1939820199	0.0603335280	0.2578382574	5.02240252*E* − 06
	0.0152114400	0.3633514726	0.3810978088	8.03072038*E* − 07
	0.1618654345	0.1097465792	0.2787430938	−1.98215448*E* − 06
	0.0056985809	0.9114653768	0.2006688671	3.21352180*E* − 08
	0.1904538879	0.2502358229	0.4452573447	6.19105762*E* − 06
	−0.1623604033	0.3089460561	0.1491746444	−9.01451453*E* − 06
	0.1864448178	0.2428992222	0.4320068557	−2.61979130*E* − 06
	−0.0449302706	0.1144916285	0.0733954587	−7.17750570*E* − 06
	0.1675935311	0.1774161896	0.3459538936	−1.27733934*E* − 05
	−0.0274959004	0.4539962587	0.4273210007	−5.20897654*E* − 06
